# Combination of αCD4 antibody and retinal antigen injection induces long-term disease control in autoimmune uveitis

**DOI:** 10.3389/fimmu.2025.1636901

**Published:** 2025-08-22

**Authors:** Dijie Qiao, Lu Zhang, Yajie Zhong, Dongmei Liu, Zilin Chen, Wai Po Chong, Jun Chen

**Affiliations:** ^1^ State Key Laboratory of Ophthalmology, Zhongshan Ophthalmic Center, Sun Yat-sen University, Guangzhou, China; ^2^ School of Chinese Medicine, Hong Kong Baptist University, Hong Kong, Hong Kong SAR, China; ^3^ Institute for Research and Continuing Education, Hong Kong Baptist University, Shenzhen, China

**Keywords:** autoimmune uveitis, immunotherapy, regulatory T cells (Treg), long-term therapy, chronic inflammation, anti-CD4 antibody

## Abstract

**Introduction:**

Autoimmune uveitis is a sight-threatening inflammatory eye disease driven by immune dysregulation. We previously introduced a therapeutic strategy involving the *in vivo* induction of retinal-antigen-specific regulatory T cells (Tregs) via αCD4 antibody injection followed by administration of the retinal self-peptide IRBP1-20, which effectively suppresses inflammation during the onset of experimental autoimmune uveitis (EAU).

**Methods:**

We evaluated the long-term therapeutic efficacy of this approach in a chronic EAU model. EAU was induced in C57BL/6 mice, and treatment was administered at each onset or relapse episode over a 28-week period. Disease progression was monitored by clinical scoring and funduscopy, with further assessment using histopathology and optical coherence tomography (OCT). Flow cytometry was employed to analyze immune cell infiltration, and RNA sequencing of ocular tissue was performed to assess gene expression changes.

**Results:**

Mice receiving aCD4 antibody and IRBP1-20 showed sustained disease control up to 28 weeks, with reduced ocular inflammation, less retinal damage on OCT, and decreased immune cell infiltration compared to untreated controls. Transcriptomic analysis revealed significant downregulation of inflammation-related genes following treatment.

**Conclusion:**

These findings support the long-term immunomodulatory effect of combining αCD4 antibody and retinal antigen injection, offering a potential strategy for managing chronic progressive autoimmune uveitis.

## Introduction

Autoimmune uveitis is clinically characterized by chronicity progressive relapses, and treatment challenges, underscoring the urgent need for effective long-term therapies. Autoimmune uveitis encompasses several common clinical subtypes, including Behçet’s disease (BD), Vogt-Koyanagi-Harada (VKH) disease, birdshot retinochoroidopathy, sarcoidosis, and sympathetic ophthalmia (1). In China, BD and VKH disease account for the majority of uveitis cases, whereas anterior uveitis and specific subtypes such as birdshot retinochoroidopathy are more prevalent in Western countries (2, 3).

Clinically, uveitis is primarily characterized by chronic inflammation, recurrent flares, and progressive disease course, posing significant challenges for long-term management. The relapsing nature of the disease is particularly pronounced, as highlighted by recent findings from the Standardization of Uveitis Nomenclature (SUN) study. Among patients with Behçet’s uveitis, only 8% exhibit an acute, monophasic presentation, whereas the majority experience acute recurrent episodes (12%) or progress to a chronic phase (72%) (2). Similarly, VKH disease follows a biphasic course: the early stage is characterized by granulomatous inflammation spanning the retinal layers, whereas the late stage leads to irreversible damage, including optic atrophy and peripheral retinal degeneration, ultimately resulting in the characteristic ‘sunset glow fundus’ appearance (3, 4). These characteristics underscore the inherently relapsing and progressive nature of uveitis, emphasizing the urgent need for targeted therapeutic strategies to mitigate disease recurrence and progression.

The experimental autoimmune uveitis (EAU) model is a widely used animal model for investigating the pathophysiology and immunological mechanisms of uveitis (5, 6). However, most published studies predominantly focus on its single-episode (monophasic) manifestation, while the chronic progression and recurrence of the disease remain underexplored (7). We have established a chronic EAU model in C57BL/6 mice, characterized by sustained retinal inflammation and progressive retinal atrophy, closely mimicking the chronic progression of human uveitis (6). Following model induction, fundoscopic examination revealed sustained retinal inflammation without spontaneous resolution, consistent with previous reports, including the chronic progressive EAU model recently described by Fan et al (8). By day 60 post-immunization, EAU mice exhibited atrophic lesions, resembling late-stage features of VKH disease. Considering that over 90% of BD-associated uveitis cases occur in the chronic phase or as acute flares, and that VKH disease often progresses to retinal atrophy in its later stages, our model reproduces key pathological features observed in chronic human uveitis- particularly the sustained inflammation and progressive retinal degeneration observed in long-standing disease. Although the PBS-treated group does not exhibit spontaneous remission or classic recurrent flares, it serves as a robust platform for studying chronic progression. Notably, recurrence-like fluctuations were only observed in the αCD4+IRBP-treated group during long-term follow-up, potentially mimicking therapeutic relapse in clinical settings. Together, these features confer translational relevance to the model for evaluating durable immunomodulatory strategies.

Regulatory T cells (Tregs) have been considered as a promising therapeutic strategy for suppressing autoimmune diseases. Extensive research, including studies from our group and others, has highlighted the significant therapeutic potential of Tregs in managing EAU (7, 9, 10). Th1 and Th17 cells are recognized as key pathogenic drivers in both uveitis and EAU (11, 12). Tregs exert their immunomodulatory effects by directly interacting with pathogenic T cells or secreting anti-inflammatory factors, thereby restoring immune homeostasis (9). In our recent study, we demonstrated the therapeutic effects of administration of low-dose αCD4 antibody (Ab) with interphotoreceptor retinoid-binding protein (IRBP)_1-20_ peptide, the pathogenic retinal antigen in uveitis, on EAU inflammation. Our mechanistic studies confirmed that low-dose αCD4 Ab induces temporary of CD4^+^ T cell apoptosis, followed by the secretion of TGF-β from phagocytic cells after the engulfment of apoptotic cells (13). These TGF- β together with the IRBP_1-20_, induces the IRBP-specific Tregs during the development of EAU (13). Given the chronic features in human autoimmune uveitis, we aim to evaluate the efficacy of this IRBP-specific Tregs immunotherapy in our chronic progressive uveitis model.

Optical coherence tomography (OCT), often referred to as “*in vivo* histology,” is a widely used ophthalmic imaging technique well-suited for the dynamic assessment of the multilayered retinal structure (14). In our previous studies, we detailed methodologies for utilizing OCT to monitor the progression of EAU (6, 15). OCT excels in the early detection of inflammatory cell infiltration, often demonstrating greater sensitivity than fundoscopy. As a non-invasive tool, OCT enables longitudinal cross-sectional visualization of retinal lesions, offering information comparable to histological analysis *in vivo*. Retinal thickness, measured via OCT, serves as a crucial marker of disease activity and strongly correlates with visual dysfunction, as assessed by electroretinography (ERG) (15). In addition, RNA sequencing (RNA-seq) has become a popular tool for studying the molecular mechanism during EAU progression. Its ability to explore the complex immune mechanisms involving both immune and ocular cells, combined with bioinformatics for hypothesis validation and pathway discovery, has made it increasingly valuable in uveitis research (16–18).

In this study, we confirmed the long-term efficacy of αCD4+IRBP therapy in treating chronic progressive EAU inflammation. Using OCT and histopathological analysis, we demonstrated the therapy’s protective effects on retinal structure, highlighting its potential for broad clinical applications. At the gene expression level, we further validated its protective impact on visual function. RNA-seq analysis identified significant transcriptional alterations across multiple immune pathways, expanding the research scope and providing valuable insights for future studies.

## Methods

### Mice

Wild-type C57BL/6 female mice (6–8 weeks old) were obtained from the Guangdong Experimental Animal Center. The mice were housed in a specific pathogen-free (SPF) facility at Zhongshan Ophthalmic Center (ZOC), Sun Yat-sen University, under a 12-hour light-dark cycle, with ad libitum access to food and water. All experimental procedures adhered to the Association for Research in Vision and Ophthalmology (ARVO) Statement for the Use of Animals in Ophthalmic and Vision Research. Ethics approval for this study was obtained from the ‘Sun Yat-sen University Animal Ethics Committee’ of ‘Sun Yat-sen University’ (No. SYXK (Yue) 2020-0058).

To ensure that the observed retinal pathology resulted from experimental manipulation rather than genetic background, all C57BL/6 mice were routinely genotyped to exclude individuals carrying the *Crb1rd8* mutation as previously described by R.R. Caspi (19). Additionally, fundoscopy was performed prior to immunization, and only animals with a normal baseline fundus appearance were included in the study.

### EAU induction and treatment protocol

EAU was induced as previously described (6, 20). Briefly, IRBP_1-20_ peptide (Hanhong Bio, Cat. 211426-18-5, Shanghai, China) was emulsified with complete Freund’s adjuvant (CFA, Sigma-Aldrich, Cat.F5881, St. Louis, MO, USA) using an ultrasonic homogenizer. The emulsion was kept on ice and immediately used for immunization. Mice were subcutaneously injected with 200 µL of emulsion (containing 150 µg IRBP_1-20_) at the base of the tail and outer thighs. To enhance the immune response, pertussis toxin (PTX, Sigma-Aldrich) was administered intraperitoneally at 0.5 µg per mouse on day0 and day 1 post immunization. For anti-CD4 and IRBP treatment, anti-CD4 antibody (αCD4 Ab, GK1.5, InVivoMAb, Cat.BE0003 Bio X Cell, Lebanon, NH, USA) was used when “onset” or “recurrence” were observed (100μg/mouse), followed by IRBP_1-20_ (3ug/mouse, i.p.) treatment every other day, 6 times in total. Mice of control group were treated with PBS at the same time. Each mice received 3-4 rounds of αCD4 Ab and IRBP therapy in the experiment. In the second treatment phase, therapy was administered only upon confirmed recurrence. The incidence rates in the treatment group were 100% for the first and second onsets, 80% for the third, and 40% for the fourth.

### Fundus and fluorescence angiography

Starting from day 11 post-immunization, disease progression was monitored. Mice were intravenously administered 1.5% pentobarbital (dose adjusted by weight), and pupils were dilated twice with 0.5% tropicamide. Hydroxypropyl methylcellulose was applied to prevent cataracts. The severity of ocular inflammation was assessed using the Micron-IV small animal retinal imaging system (Phoenix Research Laboratories, Inc., USA). Clinical scoring was performed on a standardized 0–4 scale, considering the number, type, and size of lesions, as well as the extent of inflammation, as previously described as follows (6, 20, 21): 1, Mild optic disc swelling with slightly blurred margins; few small round or linear retinal lesions; no vascular cuffing; vessels remain clearly visible; 2, Disc margins diffusely blurred with halo effect; limited inflammatory infiltrates at the optic disc; mild perivascular cuffing involving a few vessels; scattered small round and linear lesions; mild patchy scarring; 3, Prominent disc swelling with partial obscuration by lesions; moderate perivascular cuffing affecting multiple vessels; clustered round and linear lesions; moderate retinal scarring involving a substantial portion of the fundus; and 4, Marked disc atrophy with sunset halo and glassy opacity; extensive confluent perivascular cuffing; dense retinal lesions; retinal layer loss or detachment; widespread scarring occupying the majority of the fundus. Post-imaging, bacitracin ointment was applied, and mice were allowed to recover in a warm environment. For fundus fluorescein angiography, 50 μL of 1% sodium fluorescein was injected intraperitoneally, and fundus photography was initiated immediately after injection. Fundus pictures were captured continuously for the first 30 seconds, followed by one photo every 5 seconds thereafter. Leakage of retinal and choroidal vessels was observed.

### Optical coherence tomography

OCT images were obtained as previously described (6, 15, 21). Mice were anesthetized and pupils were dilated. Artificial tears (ZOC, Guangzhou, China) were used during the process. With Heidelberg Spectralis Optical Coherence Tomography System (Heidelberg, Germany), 2D-OCT images were calculated and drawn as it is shown elsewhere (22). Retinal thickness was manually measured along the horizontal axis, approximately 1 optic disc diameter away from both the nasal and temporal margins of the optic nerve head.

### Detection of eye-infiltrating cells by flow cytometry

The procedure was adapted from our previous study (23). Mice were transcardially perfused with cold PBS before tissue collection to eliminate intravascular leukocytes, ensuring that only tissue-infiltrating cells were analyzed. Following euthanasia in accordance with approved protocols, both eyes were enucleated. Extraocular tissues were removed, and the anterior segment was opened along the limbus. Intraocular fluid was collected in pre-chilled PBS. Residual tissues were minced and digested in 1 mg/mL collagenase (Sigma, Cat.C1764) at 37 °C for 50 min with gentle agitation (220 rpm). After digestion, the suspension was triturated and combined with the collected intraocular fluid. Cells were stained on ice with a panel of fluorochrome-conjugated antibodies targeting CD45, CD4, CD8, CD11b, CD11c, F4/80, Gr-1, CD19, CD3, MHC-II, and NK1.1, following standard protocols with appropriate controls. Flow cytometric analysis was performed on a BD LSRFortessa, and data were processed using FlowJo software.

### RNA-seq data processing and analysis

Total RNA was isolated from snap-frozen mouse eyeballs using a standard kit, followed by quality assessment (gel electrophoresis, Nanodrop, Agilent 2200). Libraries were prepared with the TruSeq RNA Library Prep Kit (Illumina) and sequenced as paired-end 75-bp reads on an Illumina NovaSeq platform. Raw data underwent quality filtering with FastQC.

For analysis, the top 1,000 variable genes were subjected to PCA. Differential expression analysis (DESeq2, Wald test, Bonferroni correction) identified DEGs (|log2FC| > 1, adj. p < 0.05). Functional enrichment (GO, KEGG, GSEA) was performed using clusterProfiler, with significance thresholds of adj. p < 0.05. Immune cell infiltration was estimated via mMCP-counter (24), and results were visualized as row-normalized heatmaps. Retinal cell-type deconvolution was conducted using CIBERSORT (25)with the annotated scRNA-seq dataset GSE243413 (26), based on TPM-normalized expression. Cell types with relative abundance > 0 were included for visualization.

### Real-time PCR

Total RNA was extracted from snap-frozen mouse eyeballs using TRIzol reagent (Thermo Fisher Scientific, USA) according to the manufacturer’s instructions. Complementary DNA (cDNA) was synthesized using the HiScript^®^ III RT SuperMix for qPCR Kit (Vazyme, China; Cat.R323-01).

Quantitative real-time PCR was performed using the ChamQ Universal SYBR qPCR Master Mix (Vazyme, China; Cat.Q411-02) on a Roche LightCycler^®^ 480 real-time PCR system (Roche, Switzerland). The relative expression levels of target genes were calculated using the 2^−ΔΔCt^ method as described by Schmittgen and Livak (27). Primer sequences are listed in **Table 1**.

**Table 1:** Primer sequences used in real-time qPCR test.

Gene symbol	Forward primer sequence (5′-3′)	Reverse primer sequence (5′-3′)
Serpina3n	CAACCAGAGACCCTGAGGAAGT	AGGACATCCTCCAGGCTGTAGT
Opn1sw	GTCGCCATGTTTGTGCTCTGGA	GCTTGGAGTTGAAGCGGATGCT
Rho	GAGGGCTTCTTTGCCACACTTG	AGCGGAAGTTGCTCATCGGCTT
gnat1	GCTTGTGGAAGGACTCGGGTAT	AACGCAACACGTCCTGCTCAGT
Rlbp1	GAAGATGGTGGACATGCTCCAG	CCAGGTCATCTCCGTGAACAAAG
Pde6b	TGGAGAACCGTAAGGACATCGC	TCCTCACAGTCAGCAGGCTCTT
Pde6c	GGACCAAAGACTCCAGATGGCA	GGCAATCCACTAACAAGCGTCC
Grk1	CAGATGAAGGCGACTGGCAAGA	CCAGAGACACAATGAACCTGCTG
Il17a	CAGACTACCTCAACCGTTCCAC	TCCAGCTTTCCCTCCGCATTGA
Il17f	AACCAGGGCATTTCTGTCCCAC	GGCATTGATGCAGCCTGAGTGT
Il1b	TGGACCTTCCAGGATGAGGACA	GTTCATCTCGGAGCCTGTAGTG
Cd86	ACGTATTGGAAGGAGATTACAGCT	TCTGTCAGCGTTACTATCCCGC
Ccr4	GGACTAGGTCTGTGCAAGATCG	TGCCTTCAAGGAGAATACCGCG

### Preparation of frozen eyeball sections and immunofluorescence staining

The procedure was performed as described elsewhere (28). In brief, mouse eyeballs were enucleated, rinsed in cold PBS, and fixed in 4% paraformaldehyde (PFA) at 4°C for 30 minutes. After PBS washes, the eyes were dehydrated through a 10%-20%-30% sucrose gradient (w/v in PBS, 12 h each, 4°C), embedded in OCT compound (Sakura), snap-frozen in liquid nitrogen, and stored at -80°C. Cryosections (10 μm) were prepared using a Leica CM1950 cryostat. Sections were blocked with 5% BSA (Sigma-Aldrich) for 1 h at room temperature, followed by overnight incubation at 4°C with rabbit anti-Iba1 antibody (WAKO, Cat.CAJ3125 1:400). After PBS washes, sections were incubated for 1 h with Alexa Fluor 594-conjugated anti-rabbit IgG secondary antibody (HuaBio, Cat.HA1122, 1:400). Nuclei were counterstained using DAPI-containing mounting medium (Sigma-Aldrich, Cat.P36980). Images were captured using a Zeiss LSM-880 laser confocal microscope. For quantification, Iba1^+^ microglial cells were manually counted using ImageJ software, defined as discrete Iba1^+^ signals co-localized with DAPI-stained nuclei within each field of view. The average number of Iba1^+^ cells per field was calculated across multiple sections for each group and used for statistical comparison (29).

### Paraffin-embedded eyeball sectioning and retinal layer thickness measurement

Paraffin sectioning and H&E staining were performed as previously described (30). Images were acquired using a Zeiss microscope, and quantitative analysis was conducted with Fiji software (ImageJ). As it is described elsewhere (31), Layer-specific measurements (INL, ONL) were performed within a 100 μm-wide region of interest (indicated by a red box), and the average thickness of each retinal layer was calculated based on the measured area.

### Electroretinography

The Retiport system (Roland, Consult, Germany) was used to assess the dark-adapted ERG following established protocols (6, 21). Prior to recording, mice were dark-adapted overnight, and all experimental procedures were conducted under dim red illumination to maintain the dark-adapted state. Scotopic ERG responses were recorded at stimulus intensities of 0.01, 3.0, and 10.0 cd·s/m² using the Scotopic 0.01 ERG (GF), Scotopic 3.0 ERG (GF), and Scotopic 10.0 ERG (GF) modes were applied for stimulus intensities of 0.01, 3.0, and 10.0 cd·s/m², respectively. The a-wave amplitude was measured from the baseline to the trough of the a-wave, and b-wave amplitude was measured from the trough of the a-wave to the peak of the b-wave.

### Statistical analysis

Mann-Whitney U test for unpaired data and ANOVA for multiple comparisons. All statistical results are presented as mean ± s.e.m. Statistical analysis and graphing of data were conducted using GraphPad Prism 9.0 software (GraphPad Software, San Diego, CA, USA). A significance level of p< 0.05 was considered statistically different. Symbols represent significance levels: *P < 0.05; **P < 0.01; ***P < 0.001; ****P < 0.0001.

## Results

### αCD4+IRBP therapy has long-term efficacy in suppressing EAU

**Figure 1:**
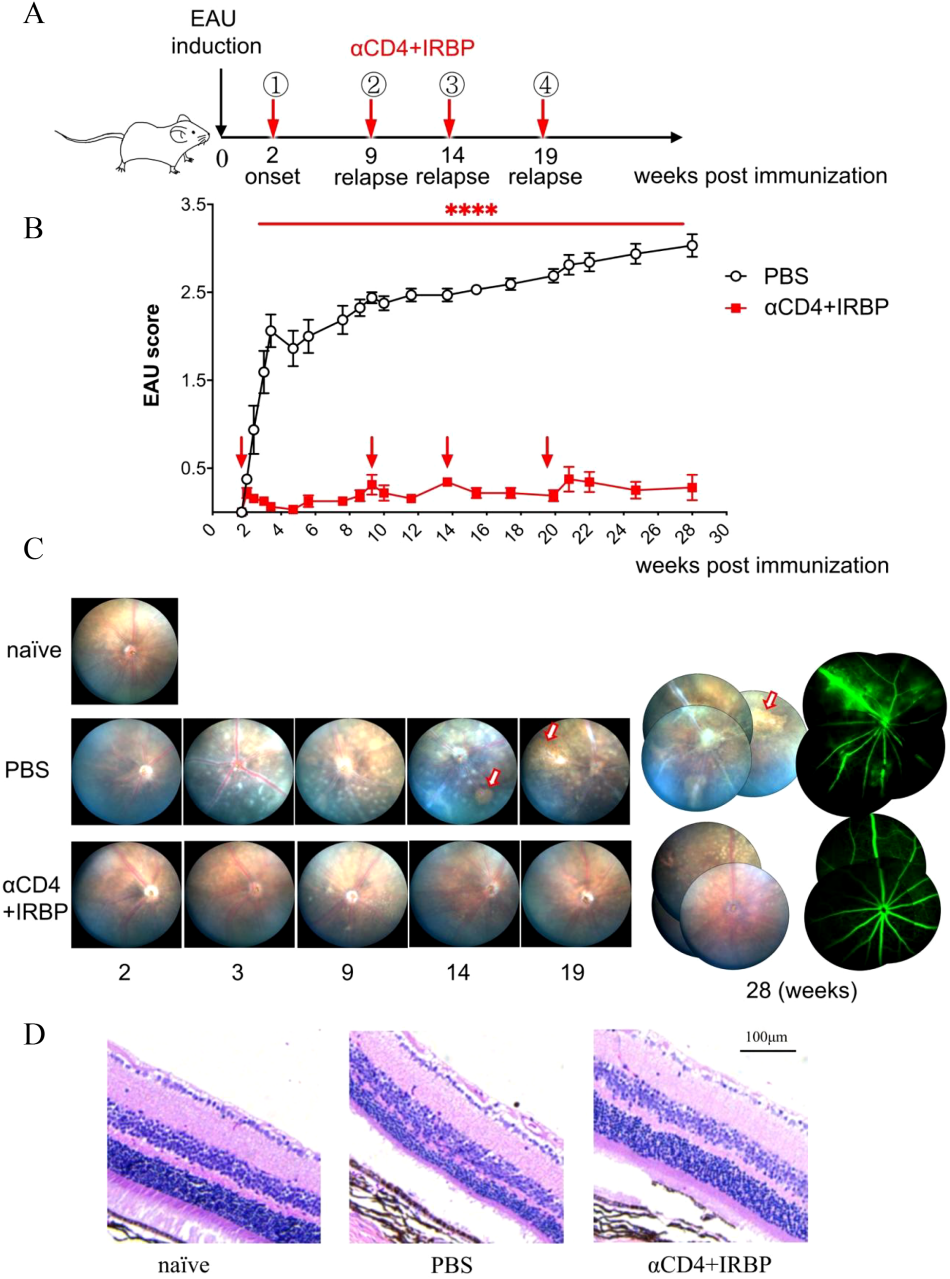
Treatment with αCD4 Ab and IRBP peptides ameliorates chronic EAU up to 28 weeks. Ocular inflammation was induced in C57BL/6 mice (female, 6-8 week) by immunization with retinal antigen IRBP peptides in CFA and pertussis toxin (PTx). **(A, B)** Experimental scheme and clinical scores of EAU in mice treated with PBS or αCD4+IRBP. Arrowhead indicates the initiation of treatment. Retinal inflammation in EAU mice was dynamically monitored with Micron-IV small animal retina imaging system and αCD4 combined with IRBP peptides was administered at disease onset (week 2 post-immunization) and at relapse time points (weeks 9, 14, and 20 post-immunization). Data are presented as mean± SEM of 9-10 mice from two individual experiments. Two-way ANOVA; linear regression curve including the 95% confidence band of the regression line. ****p<0.0001. **(C)** Representative retinal images acquired by fundoscopy (left) and fluorescein angiography (right) from onset to week 28 post-immunization. Arrows indicate areas of patchy pigment loss observed in PBS-treated eyes. **(D)** Representative images of H&E-stained paraffin sections from the eyes of EAU mice treated with αCD4 antibody and IRBP peptides. Scale bar, 100 μm.

To evaluate the effect of αCD4+IRBP therapy in chronic EAU, we induced EAU in C57BL/6 mice and performed dynamic fundus examinations over a 28-week period, undergoing four treatment cycles (**Figure 1A**). In this chronic model, optic disc inflammation (onset) was detected in wild-type mice at 2 weeks post-immunization. Consistent with therapies effective in single-phase EAU model (shown in **Supplementary Figure S1**), the first treatment was given at disease onset, effectively alleviating symptoms in the treatment group. Prior to the long-term study, we conducted a pre-experiment to verify that a single injection of αCD4 antibody is sufficient to induce transient CD4^+^ T cell depletion, which is the intended mechanism of our protocol, as previously reported (13). In contrast, the control group exhibited no recovery and progressed to chronic lesions characterized by linear changes (**Figure 1B**). Chronic retinal vasculitis episodes were observed at weeks 9, 14, and 19 post-immunizations. Treatment was administered at each relapse, effectively controlling the inflammation in the treatment group (**Figure 1B**). In contrast, the control group exhibited persistent inflammation, characterized by new vascular leakages superimposed on pre-existing lesions. Sustained retinal inflammation in the control group, without resolution, led to progressive structural damage, characterized by linear lesions, retinal thinning, and irregular areas of RPE depigmentation (i.e., patchy pigment loss), as visualized in fundus pictures (**Figures 1C, D**).

At week 28 post-immunization, fundus fluorescein angiography (FFA) was performed. Representative images from the control group revealed extensive vascular leakage (10 o’clock), zigzag vascular alterations (8 o’clock), and vascular filling defects (6 o’clock) (**Figure 1C**). Histopathological analysis of H&E-stained sections revealed that in the control group of the chronic EAU model, retinal architecture was disrupted, layer boundaries were indistinct, and overall retinal thickness was significantly reduced (**Figure 1D**). Conversely, the treatment group exhibited intact and well-defined retinal structures, comparable to those of naïve (healthy control mice) in an age-matched cohort.

### αCD4+IRBP administration preserves retinal structure

**Figure 2:**
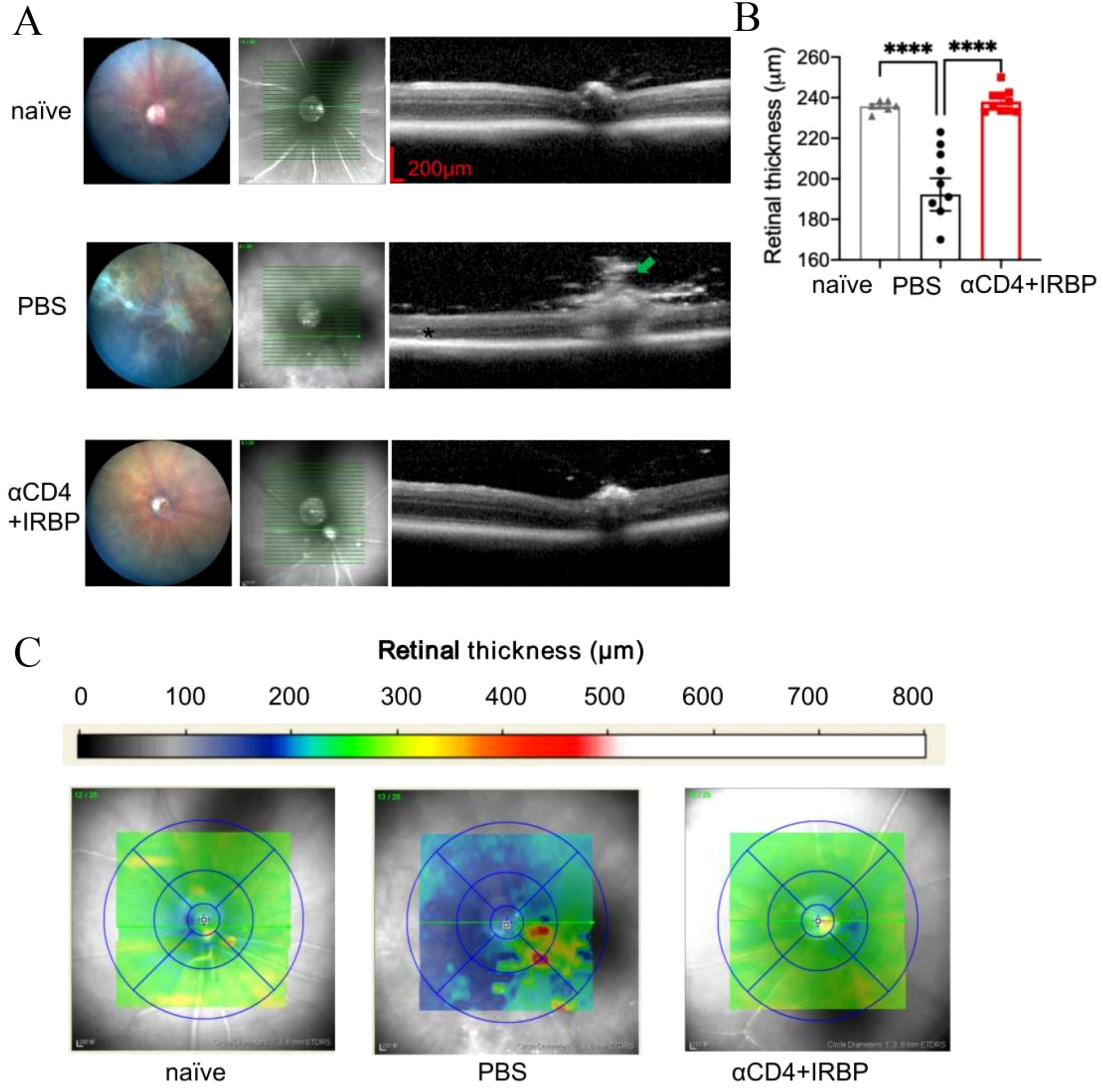
OCT analysis of retinal structure and thickness topography in EAU mice. **(A)** Representative fundus images and OCT cross-sectional views through the optic disc from naïve, EAU, and treatment groups. At 28 weeks, untreated EAU mice exhibited prominent vasculitis (green arrow), retinal atrophy (red arrows), and severe cellular infiltrates (*). **(B)** Quantitative analysis of retinal thickness measured manually at 1 PD from the optic disc approximately 1 optic disc diameter on both sides of the optic disc. Data are presented for naïve (n=6), PBS (n=9), and αCD4+IRBP treated groups (n=10). **(C)** Retinal thickness maps generated using automated calculations from the Heidelberg Spectralis OCT system. Yellow-green regions indicate normal retinal thickness, whereas blue regions represent retinal thinning, and red regions indicate localized thickening. ****p < 0.0001.

To precisely evaluate histological differences in the retinas of the treatment group and control group, we utilized OCT for cross-sectional imaging of the mouse fundus. retinal signal was abtained by the Heidelberg Spectralis OCT system and retinal thickness was measured at one optic disc diameter from the optic nerve head. **Figure 2A** illustrates the schematic of the OCT scanning location along with representative retinal thickness images. Statistical analysis showed that retinal thickness in the control group was significantly reduced compared to healthy mice, whereas the αCD4+IRBP-treated group maintained retinal thickness at levels comparable to healthy controls (**Figure 2B**). Moreover, these changes were observed throughout different regions of the fundus, including the optic disc, ciliary body, and peripheral retina (**Supplementary Figure S2**).

Retinal thickness topography maps provide an intuitive visualization of these findings (**Figure 2C**). In healthy mice, retinal thickness ranged from 220 to 260 µm (green to yellow), with the majority of αCD4+IRBP-treated mice exhibiting values within this range. In contrast, retinal thickness in the PBS-treated control group ranged from 160 to 220 µm (blue), with widespread retinal thinning. In some regions, focal hyperreflective foci (HRF) were observed, which may reflect inflammation-related changes such as activated microglia, extracellular matrix remodeling, or proteinaceous deposits. Such OCT features have been described in previous studies of chronic uveitis (8, 32) and are considered indicative of underlying inflammatory pathology.

ERG results indicated that both PBS-treated and αCD4+IRBP-treated mice exhibited reduced b-wave and a-wave amplitudes compared to naïve controls, with the reduction being more pronounced in the PBS group (**Supplementary Figure S3**). These findings visually demonstrate the protective effect of αCD4+IRBP therapy on retinal structure and integrity.

### αCD4+IRBP therapy modulates pathways involved in visual function and immune response

**Figure 3:**
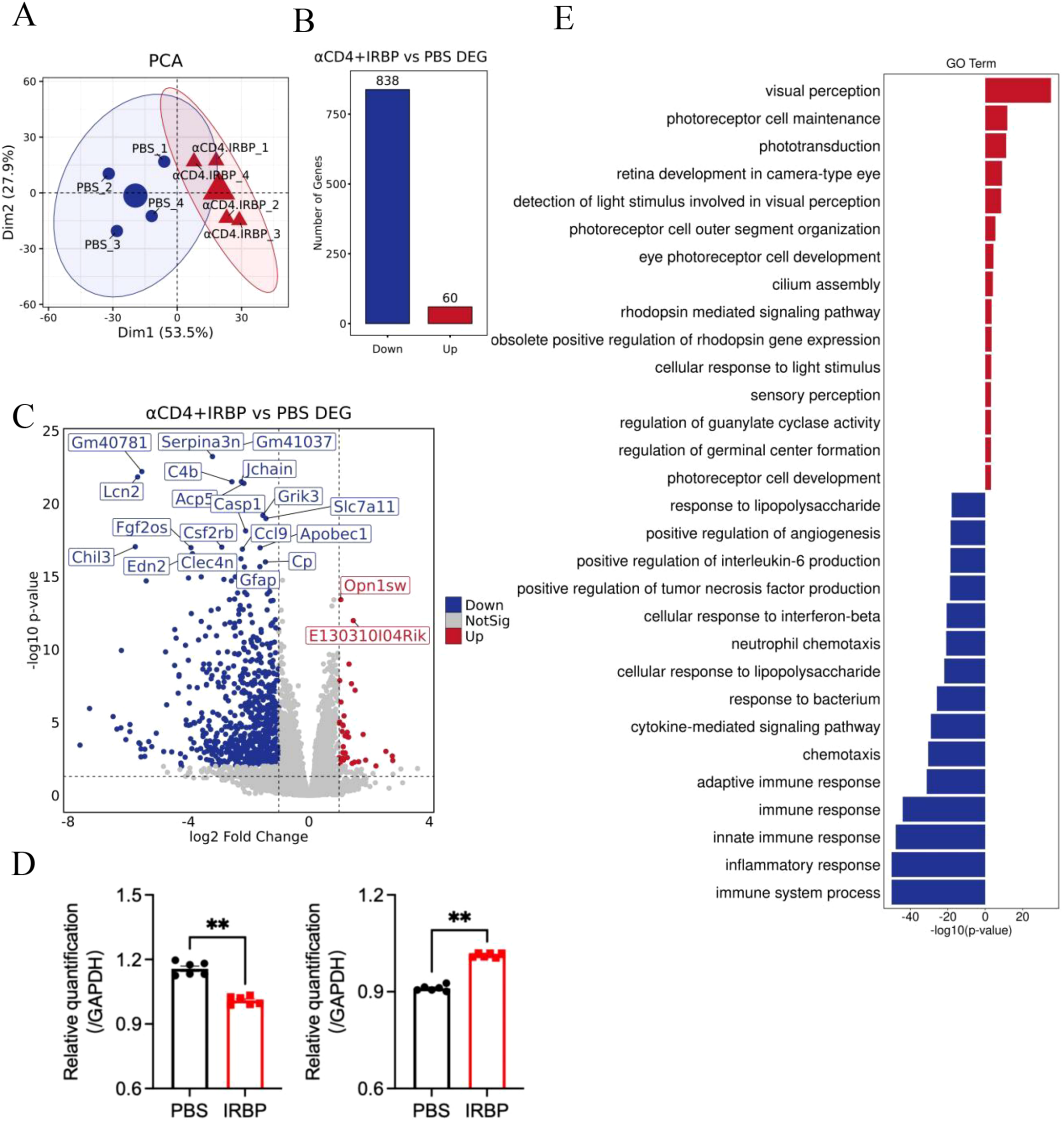
Impact of αCD4+IRBP therapy on gene expression and signaling pathways. **(A)** Principal component analysis (PCA) of gene expression profiles across groups, demonstrating distinct clustering patterns. **(B)** Differentially expressed genes (DEGs) identified following αCD4+IRBP therapy. Blue dots represent downregulated genes, while red dots represent upregulated genes. **(C)** Volcano plot depicting the significance (-log10 adjusted p-value) and magnitude (log2 fold change) of DEGs. **(D)** Validation of RNA-seq findings by qPCR, confirming significant downregulation of Serpina3n (left) and Opn1sw (right) in the treatment group (both p < 0.01). **(E)** Gene Ontology (GO) enrichment analysis of biological pathways. Blue bars represent significantly downregulated pathways following treatment, while red bars represent significantly upregulated pathways. **p<0.01.

To investigate the molecular mechanisms underlying the protective effects of αCD4+IRBP therapy in EAU mouse model, we performed bulk RNA-seq analysis on eye tissues collected at week 28 post-treatment from both the treatment and PBS-treated control groups. Principal Principal Component Analysis (PCA) of RNA-seq data revealed greater inter-individual variability in the PBS-treated control group, likely due to repeated disease episodes (3-4) and accumulated inflammation. In contrast, samples from the αCD4+IRBP treatment group showed minimal differences among individuals after disease progression was controlled by the treatment (**Figure 3A**). Compared to the PBS-treated control group, differential gene expression analysis 838 downregulated and 60 upregulated genes in the αCD4+IRBP-treated group (**Figure 3B**).

A volcano plot visualizing differentially expressed genes (DEGs) following αCD4+IRBP therapy is shown in **Figure 3C**. Notably, serpina3n and opn1sw were among the top downregulated genes, with adjusted p-values < 0.01 and absolute log2 fold changes > 2. To validate these transcriptomic findings, we performed qPCR analysis, which confirmed the significant downregulation of both serpina3n and opn1sw in the treatment group (p < 0.01, **Figure 3D**). A summary of the top 20 downregulated DEGs, ranked by false discovery rate and fold change, is provided in **Table 2**. Gene Ontology (GO) enrichment analysis revealed that upregulated genes were predominantly involved in visual function-related pathways, including visual perception, photoreceptor cell maintenance, and phototransduction pathways. These findings indicate that αCD4+IRBP therapy plays a crucial role in maintaining retinal integrity and preserving visual function. Conversely, downregulated genes were predominantly associated with immune response and inflammatory pathways, further supporting the therapy’s efficacy in suppressing pathological immune activation (**Figure 3D**). Meanwhile, KEGG pathway analysis of all DEGs corroborated the GO findings. The phototransduction pathway was the most significantly upregulated, highlighting its role in converting absorbed photons into molecular signals. Among the top 10 downregulated pathways, the majority were associated with inflammation (**Table 3**).

**Table 2:** Top 20 Differentially expressed genes (DEGs) Iidentified in bulk sequencing of ocular tissue of αCD4+IRBP treatment.

Gene symbol	FDR*	log2FC†	Description
Gm41037	8.27271E-25	-2.298363487	Predicted gene 41037
Serpina3n	5.74317E-24	-3.203146001	Serine protease inhibitor A3N
Gm40781	5.09517E-23	-5.548686784	Predicted gene 40781
Lcn2	1.41657E-22	-5.689246185	Neutrophil gelatinase-associated lipocalin
C4b	1.83957E-22	-2.558550367	Complement component 4B (Childo blood group)
Jchain	1.83957E-22	-2.242756128	Joining chain of multimeric immunoglobulin
Acp5	2.61546E-22	-2.158397757	Tartrate-resistant acid phosphatase type 5
Grik3	5.71896E-20	-1.529473931	Glutamate receptor ionotropic, kainate 3
Slc7a11	8.44163E-20	-1.428828704	Putative uncharacterized protein
Casp1	6.099E-19	-2.10323327	Caspase-1
Csf2rb	5.7619E-18	-2.894770749	Cytokine receptor common subunit beta
Chil3	8.0578E-18	-5.766748717	Chitinase-like protein 3
Apobec1	9.2488E-18	-1.622710919	C->U-editing enzyme APOBEC-1
Fgf2os	9.33238E-18	-3.917604802	Putative uncharacterized protein
Ccl9	9.49028E-18	-2.210374664	C-C motif chemokine 9
Edn2	2.61051E-17	-3.870113152	Endothelin-2
Mmp12	3.74582E-17	-2.258786526	Macrophage metalloelastase
Cp	7.01384E-17	-1.446118336	Ceruloplasmin, isoform CRA_f
Clec4n	1.28073E-16	-2.669924474	C-type lectin domain family 4, member n, isoform CRA_a
Gfap	1.4194E-16	-1.628788935	Glial fibrillary acidic protein

*FDR, False Discovery Rate; †FC, Fold Change.

**Table 3:** KEGG pathways of top 10 upregulated and top 10 downregulated pathways.

Upregulated pathways	FDR*	No. of genes	Gene
Phototransduction	2.37718E-20	14	Cnga1、Cngb1、Gnat1、Gnb1、Gngt1、Grk1、Gucy2e、Gucy2f、Pde6a、Pde6b、Pde6g、Rcvrn、Rho、Slc24a1
Metabolic pathways	0.000474318	31	Adcy6、Agpat3、Alox15、Alpl、Arg2、Cds1、Cyp4a12a、Dgke、Elovl2、Elovl4、Eno3、Gucy2e、Gucy2f、Hdc、Hk2、Ipmk、Nt5e、Pde6a、Pde6b、Pde6g、Pfkfb2、Plcd3、Plpp2、Pnpla3、Prdm9、Rdh12、、dh8、Suv39h2、Tmlhe、Tpo、Uckl1
Purine metabolism	0.01175635	7	Adcy6、Gucy2e、Gucy2f、Nt5e、Pde6a、Pde6b、Pde6g
Glycerolipid metabolism	0.092135648	4	Agpat3、Dgke、Plpp2、Pnpla3
Cholinergic synapse	0.107362876	5	Adcy6、Gnb1、Gnb5、Gngt1、Kcnj14
Serotonergic synapse	0.143436613	5	Alox15、Gnb1、Gnb5、Gngt1、Htr5a
Apelin signaling pathway	0.143436613	5	Adcy6、Gnb1、Gnb5、Gngt1、Mef2c
GABAergic synapse	0.143436613	4	Adcy6、Gnb1、Gnb5、Gngt1
Morphine addiction	0.143436613	4	Adcy6、Gnb1、Gnb5、Gngt1
ABC transporters	0.143436613	3	Abca13、Abca4、Abcg4
Downregulated Pathways			
Phototransduction	2.37718E-20	14	Cnga1、Cngb1、Gnat1、Gnb1、Gngt1、Grk1、Gucy2e、Gucy2f、Pde6a、Pde6b、Pde6g、Rcvrn、Rho、Slc24a1
Metabolic pathways	0.000474318	31	Adcy6、Agpat3、Alox15、Alpl、Arg2、Cds1、Cyp4a12a、Dgke、Elovl2、Elovl4、Eno3、Gucy2e、Gucy2f、Hdc、Hk2、Ipmk、Nt5e、Pde6a、Pde6b、Pde6g、Pfkfb2、Plcd3、Plpp2、Pnpla3、Prdm9、Rdh12、、dh8、Suv39h2、Tmlhe、Tpo、Uckl1
Purine metabolism	0.01175635	7	Adcy6、Gucy2e、Gucy2f、Nt5e、Pde6a、Pde6b、Pde6g
Glycerolipid metabolism	0.092135648	4	Agpat3、Dgke、Plpp2、Pnpla3
Cholinergic synapse	0.107362876	5	Adcy6、Gnb1、Gnb5、Gngt1、Kcnj14
Serotonergic synapse	0.143436613	5	Alox15、Gnb1、Gnb5、Gngt1、Htr5a
Apelin signaling pathway	0.143436613	5	Adcy6、Gnb1、Gnb5、Gngt1、Mef2c
GABAergic synapse	0.143436613	4	Adcy6、Gnb1、Gnb5、Gngt1
Morphine addiction	0.143436613	4	Adcy6、Gnb1、Gnb5、Gngt1
ABC transporters	0.143436613	3	Abca13、Abca4、Abcg4

### RNA-seq shows visual function protection

**Figure 4:**
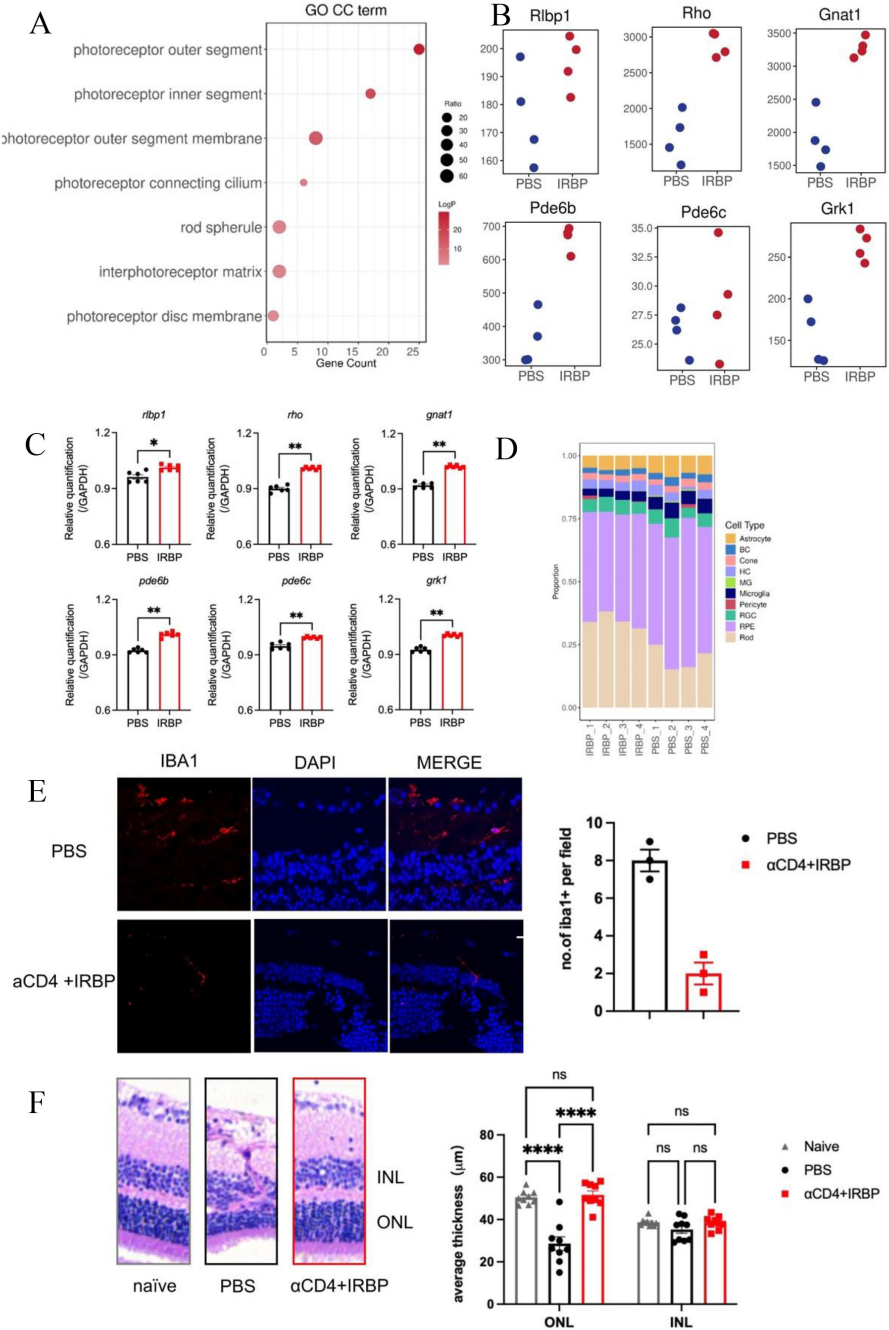
αCD4+IRBP therapy preserves retinal architecture and enhances vision-related pathways. **(A)** GO enrichment analysis of cellular component (CC) terms, identifying key pathways associated with retinal structure and function preserved by αCD4+ IRBP treatment. **(B)** Increased expression of representative genes involved in visual signaling pathways following αCD4+IRBP therapy. **(C)** qPCR validation confirming the upregulation of vision-related genes identified in **(B)**, including *Rlbp1, Rho, Gnat1, Pde6b, Pde6c, and Grk1 *;**(D)** Deconvolution analysis of retinal cell type proportions in untreated (PBS1-4) and αCD4+ IRBP treated (IRBP1-4) retinal samples. Transcriptomic data deconvoluted using the CIBERSORT algorithm, based on characteristic gene signatures of retinal cell types annotated from the mouse single-cell dataset GSE243413. transcripts per million (TPM) expression matrices were used as input, and only cell types with a total relative abundance greater than zero were included for visualization. **(E)** Representative images and quantitative analysis of immunofluorescence staining on frozen eye sections at week 28 post-immunization. Iba1^+^ cells were manually counted using ImageJ based on discrete Iba1^+^ signals co-localized with DAPI-stained nuclei. Data are shown for PBS (n, 3) and αCD4+IRBP-treated groups (n, 3). For each group, one representative mouse was selected, from which three non-adjacent sections were analyzed. One field of view was evaluated per section. Scale bar, 10 μm. **(F)** Representative images and quantitative analysis of H&E-stained paraffin sections of the eye at week 28 post-immunization. For each mouse, one eye was selected for sectioning. Three non-adjacent sections were obtained, and three random fields per section were analyzed. Retinal layer thickness (INL and ONL) was measured within a 100 μm -wide region of interest in each field. Each data point represents the average of 9 fields of view from one eye. Data are shown for naïve (n=9), PBS (n=9), and αCD4+IRBP-treated groups (n=9). *p < 0.05; **p < 0.01; ****p < 0.0001.

GO cellular component (GO CC) enrichment analysis revealed that αCD4+IRBP therapy significantly enriched multiple pathways associated with photoreceptor structures, such as the photoreceptor outer segment, inner segment, and outer segment membrane. These findings suggest that αCD4+IRBP therapy exerts its protective effects by preserving these critical photoreceptor structures (**Figure 4A**). To further assess the impact of treatment on phototransduction, we selected key genes from the phototransduction pathway (*Rho, Gnat1, Rlbp1, Pde6b, Pde6c, and Grk1*). RNA-seq analysis showed that these genes were significantly upregulated in the treatment group (**Figure 4B**). To validate these findings, we performed qPCR, which confirmed the elevated expression levels of all six genes in the treatment group compared to controls (p < 0.01, **Figure 4C**), supporting the protective effect of the therapy on retinal function.

Additionally, deconvolution analysis of retinal cell type proportions across different samples showed that the proportion of Rod cells was generally higher in the treatment group, with a clear increase compared to the control group. The proportion of Cone cells showed less variation between the treatment and untreated (PBS) group, remaining relatively stable in the treatment group while fluctuating in the control group. The proportion of microglia was consistently lower in the treatment group than in the PBS group, suggesting that αCD4+IRBP therapy may improve the retinal microenvironment by alleviating inflammation (**Figure 4D**). Consistent with this, immunofluorescence analysis further demonstrated a reduction in Iba1^+^ microglia in the treatment group, as quantified by manual cell counting based on Iba1 and DAPI co-localization (**Figure 4E**). Quantitative analysis of representative H&E-stained retinal paraffin sections (width: 100 μm) revealed that ONL thickness in the treatment group was preserved at levels comparable to non-immunized controls, whereas a significant thinning was detected in the PBS group. No significant differences in INL thickness were observed among the groups, further supporting the conclusion of substantial rod cell loss in the PBS group (**Figure 4F**).

### RNA-seq reveals downregulation of immune-related pathways

**Figure 5:**
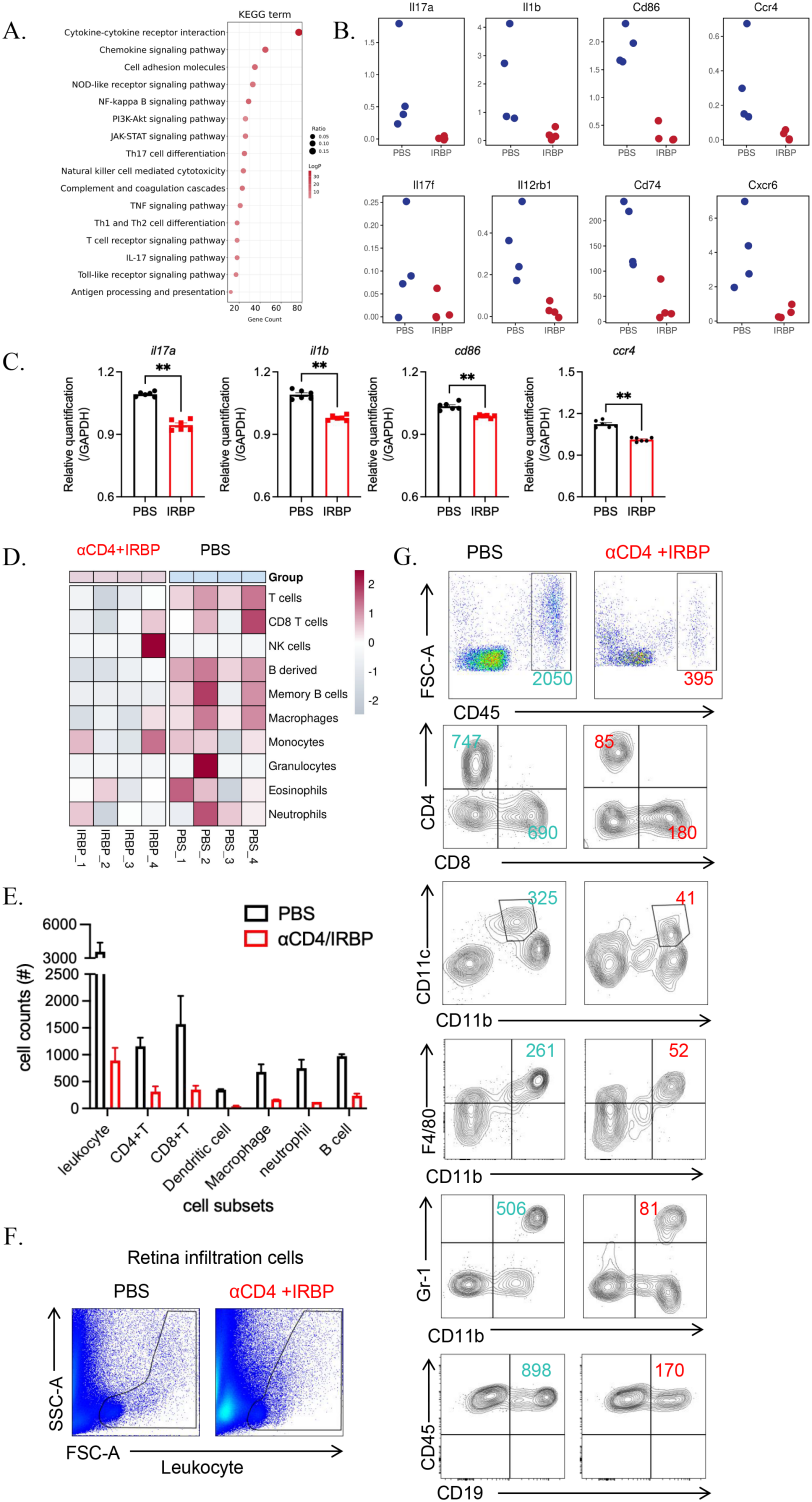
αCD4+IRBP therapy suppresses immune responses and inflammatory cell infiltration. **(A)** KEGG pathway analysis illustrating representative immune and inflammatory pathways significantly downregulated following αCD4+IRBP therapy. **(B)** Representative inflammatory mediators—including cytokines and chemokines-among the downregulated differentially expressed genes (DEGs) after treatment. **(C)** qPCR validation confirms significant downregulation of *Il17a*, *Il1b*, *Cd86*, and *Ccr4*. **(D)** Changes in immune cell composition estimated using the mouse Microenvironment Cell Population Counter (mMCP-counter) algorithm. Red indicates increased immune cell populations, blue indicates decreased populations, and white represents no significant change, reflecting the immune cell dynamics modulated by αCD4+IRBP therapy. **(E)** Quantification of absolute numbers of total leukocytes (CD45+), CD4+ and CD8+ T cells, dendritic cells (CD11c^+^CD11b^+^), macrophages (CD11b^+^F4/80^+^), neutrophils (CD11b^+^Gr-1^+^), and B cells (CD45^+^CD19^+^) in ocular tissues, as determined by flow cytometry. **(F)** Representative flow cytometry plots showing infiltrating inflammatory cell populations isolated from whole eyeballs of EAU mice. **(G)** Comparison of infiltrating immune cell distribution between PBS-treated and αCD4+IRBP-treated mice based on flow cytometry analysis. **p<0.01.

KEGG pathway enrichment analysis identified significant enrichment of downregulated genes in multiple immune-related pathways (**Figure 5A**), including Th17 cell differentiation, Th1/Th2 cell differentiation, and T cell receptor (TCR) signaling pathways. These findings align with our previous results, demonstrating that αCD4 Ab combined with IRBP therapy suppresses the pathological differentiation of Th1 and Th17 cells and inhibits T cell-mediated pro-inflammatory pathways. We also identified several key inflammation-associated genes that were significantly downregulated in the treatment group, including pathogenic T cell-related factors (*Il17a, Il17f, Il12rb1*), innate immune cell-associated markers (*Il1b, Cd74, Cd86*), and chemokine receptors (*Ccr4, Cxcr6*) (**Figure 5B**). These genes are crucial regulators of inflammatory signaling, cytokine regulation, and chemokine-mediated immune responses, highlighting the anti-inflammatory effects of αCD4+IRBP therapy. To validate the transcriptomic findings, we performed qPCR, which confirmed the downregulation of representative inflammatory genes (*Il17a, Il1b, Cd86*, and *Ccr4*) in the treatment group (p < 0.01, **Figure 5C**).

In addition, increased expression of the Treg cell marker Foxp3 and related functional markers, including *Icos, Ctla4, and Il10*, was observed in the PBS group (shown in **Supplementary Figure S4A**). Given that this measurement was performed 8 weeks after the last treatment (week 20), the findings were not unexpected. Indeed, within just 7 weeks, the expression levels of Tregs in the treatment group, as assessed by flow cytometry on day 60, had already returned to levels comparable to those of the PBS group (shown in **Supplementary Figure S4B**), highlighting the necessity for a second round of treatment.

Using the mouse Microenvironment Cell Population Counter (mMCP-counter) (18), we assessed the types of immune cells present in the eyeballs. The results revealed increased infiltration of granulocytes, monocytes/macrophages, B cells, and T cells in the eyes of untreated EAU mice (**Figure 5D**). These findings were further validated by flow cytometry (**Figure 5E**), which demonstrated a reduction in CD4^+^ and CD8^+^ T cells, dendritic cells, macrophages, neutrophils, and B cells following treatment (**Figures 5F, G**).

## Discussion

Our study demonstrates that low- dose αCD4 Ab combined with IRBP therapy is effective in treating chronic, progressive EAU mouse model. Compared to untreated EAU mouse models, the treated mice exhibited a significant reduction in retinal inflammation and less inflammatory cell infiltration. In the later stage, retinal thickness of untreated EAU mice significantly decreased the treated group stable and close to baseline levels. Transcriptomic analysis demonstrated downregulation of inflammatory pathways post-treatment, and both convolutional cloud analysis and flow cytometry confirmed a decrease in immune cells. Pathways related to visual signal transduction were upregulated, and the proportion of core retinal cells responsible for visual function were preserved.

The induced EAU model on the C57BL/6 background remains the most widely used and classical model for studying uveitis. Traditionally, this model has been considered self-limiting, with inflammation gradually subsiding by week 4, and peak clinical scores typically not exceeding 2.5, as originally reported by the R.R. Caspi laboratory in the United States. However, using the same immunization protocol, we (ZOC, China) and several other laboratories have observed a markedly different disease course, where inflammation follows a slow progressive trajectory without spontaneous resolution. We speculate that this discrepancy may be largely attributed to differences in microbiological environments across animal facilities. In addition to persistent inflammation leading to RPE loss and multifocal retinal atrophy, we also observed chronic inflammatory episodes specifically in the optic disc region, a feature independently validated by the team of Ilva D. Rupenthal in New Zealand (33). Given such a complex and protracted disease pattern, conventional clinical scoring focused solely on “active inflammation” is no longer sufficient. An appropriate scoring system must account for both inflammatory activity and structural damage to accurately reflect disease severity. In our previous publications, we evaluated EAU severity using a combination of OCT imaging, electroretinography (ERG), and histopathological analysis. The scoring characteristics of wild-type mice under similar conditions are described therein. In the present study, we applied a long-term fundus-based scoring system to assess the therapeutic effect of αCD4+IRBP treatment. In the early disease phase (weeks 2-4), fundus scores were generally in the range of 0.5-2. By week 28, however, untreated wild-type mice displayed severe retinal pathology- including retinal vasculitis, vascular occlusion, multifocal atrophy, loss of retinal layers, and retinal detachment- with fundus scores reaching 2.5-4.

Low-dose antibodies inducing apoptosis in specific cells have been a well-established approach since the late 20th century (34). Researchers discovered that certain antibodies could trigger programmed cell death (apoptosis) by binding to specific antigens on the cell surface (35). Subsequent studies have further explored the mechanisms underlying this process, advancing the use of antibody-based therapies for Type I diabetes and autoimmune diseases (36–38). The combination of αCD4 antibodies and autoantigen-induced Treg therapy shows promise for treating autoimmune diseases by temporarily depleting CD4+ T cells and promoting antigen-specific Tregs. In models of experimental autoimmune encephalomyelitis (EAE) (37) and EAU (13), this approach has demonstrated strong therapeutic effects. αCD4 Ab induce apoptosis in pathogenic CD4+ T cells, and phagocytic cells that engulf these apoptotic cells release immunosuppressive cytokines, such as TGF-β and IL-10. These cytokines, in conjunction with the autoantigen, promote the generation of Tregs, which suppress pathogenic Th17 and Th1 responses while preserving anti-tumor and anti-infection immunity. Building on previous research, this study further confirms the efficacy of αCD4 antibody combined with autoantigen therapy in chronic progressive disease models, providing additional support for the clinical translation of this therapy.

The pathogenesis of uveitis is complex, involving multiple immune cells across different stages of the disease (7, 12, 39, 40). Beyond the well-documented roles of Th1 and Th17 T cells (12), immune cells such as dendritic cells (41) and natural killer cells (42, 43), as well as CD8+ T cells (16, 44), also play significant roles in disease progression. Consistent with previous findings, our study observed higher T cell abundance in the PBS group, which was reduced in the IRBP treatment group. Flow cytometry further confirmed a marked increase in CD8+ T cells, alongside CD4+ T cells and their memory subsets. Existing literature suggests that CD8+ T cells play a more “auxiliary” role in the pathogenesis of EAU, progressively infiltrating ocular tissues as the disease progresses (44, 45). However, the complex and multifaceted role of CD8^+^ T cells necessitates further investigation to fully understand their precise contributions (16). In this study, macrophages and monocytes were abundant in both groups, but macrophage abundance significantly decreased in the IRBP treatment group compared to PBS controls, as confirmed by flow cytometry. Macrophages, which can polarize into pro-inflammatory M1 and anti-inflammatory M2 subtypes, are predominantly of the M1 phenotype in BD, contributing to a pro-inflammatory environment (41, 46). These findings indicate that the combination of αCD4 Ab and IRBP therapy reduces ocular damage in chronic EAU mice by suppressing the infiltration and differentiation of B cells and macrophages.

In addition to the reduction in immune cell infiltration, the protection of functional cellular structures is also a key aspect of EAU treatment. This study uses pathological slides, OCT, and RNA-seq from multiple perspectives to confirm that the therapy can preserve retinal structure and function in chronic EAU. By GO analysis, we found that the top three upregulated terms were Photoreceptor Outer Segment, photoreceptor inner Segment, and Photoreceptor Outer Segment membrane. The outer segment is the primary region where photoreceptor cells receive and convert light signals. Photons striking photoreceptors such as rhodopsin trigger photochemical reactions, leading to conformational changes in photoreceptor proteins and initiating a series of signal transduction processes, ultimately generating electrical signals (47). The upregulation of the Photoreceptor Outer Segment pathway is consistent with the preservation of the phototransduction pathway. The photoreceptor inner segment is responsible for energy supply, substance synthesis, and supporting the outer segment’s functions, with its upregulation also aligning with the phototransduction pathway (48, 49). Additionally, other upregulated pathways in the Cellular Component category include cell projection, photoreceptor connecting cilium, ciliary basal body, interphotoreceptor matrix (extracellular matrix surrounding photoreceptors or between them and the RPE), rod spherule, and heterotrimeric G-protein complex, indicating that these cellular structures or matrix components were preserved following treatment, without being degraded due to chronic inflammatory damage.

Our study presents several novel contributions. First, although the short-term efficacy of αCD4 Ab and IRBP therapy has been previously established, we specifically addressed its long-term therapeutic effects and late-stage impact. Notably, we identified a pattern of periodic recurrence during treatment, which coincided with the Tregs infiltration cycle, providing important insights for the clinical translation of this therapy. Second, although multiple research groups from different countries have independently reported the chronic and relapsing nature of the induced EAU model, to our knowledge, we are the first to explore therapeutic strategies using this model. We hope our work will offer new perspectives and inspiration for other researchers in the field of ophthalmology. Finally, RNA-seq analysis at the late stage of the induced EAU model revealed pathological changes that may highlight novel therapeutic targets for future investigation.

Our study has certain limitations. Firstly, our long-term dynamic follow-up using ERG failed to yield the expected conclusions. In our previous experiments (13), we demonstrated that during the initial peak phase of EAU (day 28), ERG recordings showed no significant differences in a- and b-wave amplitudes between the two groups. However, by day 60, a difference in b-wave amplitude emerged. In the current study, ERG assessment at week 23 revealed that a- and b-wave amplitudes had again returned to comparable levels between the groups. Considering the destructive impact of EAU on retinal function as assessed by ERG, we speculate that repeated episodes of EAU, although controlled each time, induced irreversible damage. Therefore, we propose that in future clinical applications of αCD4 Ab and IRBP therapy, prophylactic, periodic administration may be more beneficial than initiating treatment only after the onset of symptoms. Despite the lack of ideal longitudinal ERG data in this study, we revealed structural alterations using OCT and detected changes in neural signaling pathways through RNA analysis, confirming that αCD4 therapy still exerts a protective effect on visual function. Additionally, we observed that late-stage EAU exhibited diverse pathological features, including retinal atrophy (dry course) and inflammatory cell infiltration (wet course); however, we did not perform subtype classification, which warrants further investigation. Moreover, the mechanisms underlying the migration of systemic Tregs induced by αCD4 therapy to the ocular tissue remain unclear and require further dedicated research. Notably, RNA-seq analysis at week 28 revealed a localized upregulation of ccl20 in the treatment group (**Supplementary Figure S4A**); however, whether this chemokine plays a critical role in Tregs recruitment remains to be determined.

This study, building on prior research using single-phase models of αCD4 Ab combined with IRBP therapy, incorporates pathological techniques (such as histopathology and OCT) and high throughput sequencing to demonstrate that this therapy effectively controls chronic progressive EAU inflammation. It achieves this by reducing inflammatory cell infiltration and preserving the retina’s normal physiological structure and function. Notably, in the chronic model, the infiltration of B cells and macrophages, along with the maintenance of visual transduction pathways, including the *Opn1sw* gene, may serve as key targets for future research on chronic uveitis.

## Data Availability

The raw sequencing data generated in this study have been deposited in the Genome Sequence Archive (GSA) of the National Genomics Data Center, Chinese Academy of Sciences, under the project accession number PRJCA044626. Other relevant data supporting the findings of this study are included in the article and **Supplementary Materials** or are available from the corresponding author upon reasonable request.
