# Supplementary material for: Combination of αCD4 antibody and retinal antigen injection induces long-term disease control in autoimmune uveitis
Source: Front Immunol. 2025 Aug 22;16:1636901. doi: 10.3389/fimmu.2025.1636901 (PMC12411532; doi:10.3389/fimmu.2025.1636901)
Supplement: Supplementary file 1 [file Presentation1.pptx]

## Slide 1
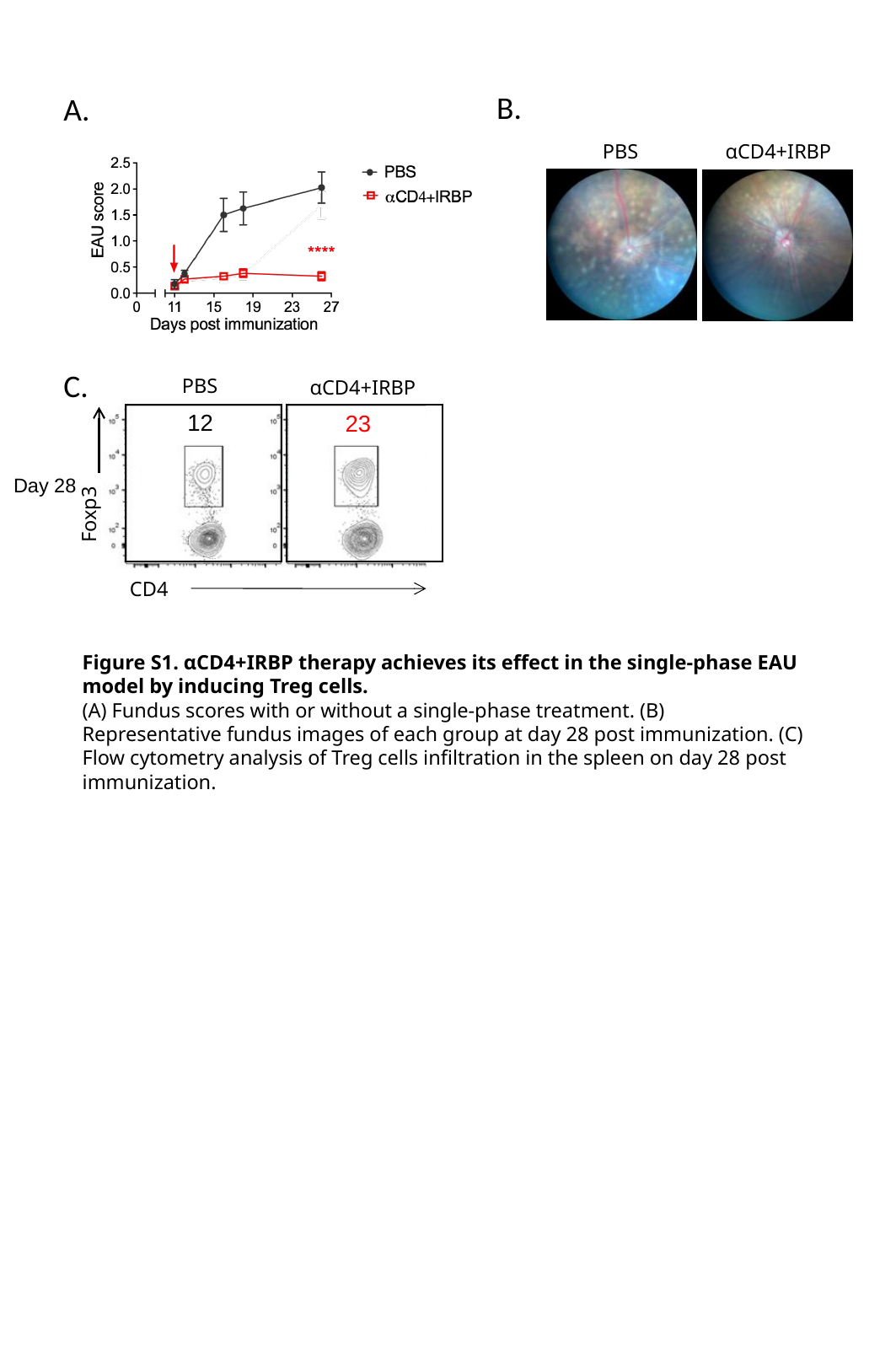

B.
A.
PBS
αCD4+IRBP
C.
PBS
αCD4+IRBP
12
23
Foxp3
CD4
Day 28
Figure S1. αCD4+IRBP therapy achieves its effect in the single-phase EAU model by inducing Treg cells.
(A) Fundus scores with or without a single-phase treatment. (B) Representative fundus images of each group at day 28 post immunization. (C) Flow cytometry analysis of Treg cells infiltration in the spleen on day 28 post immunization.

## Slide 2
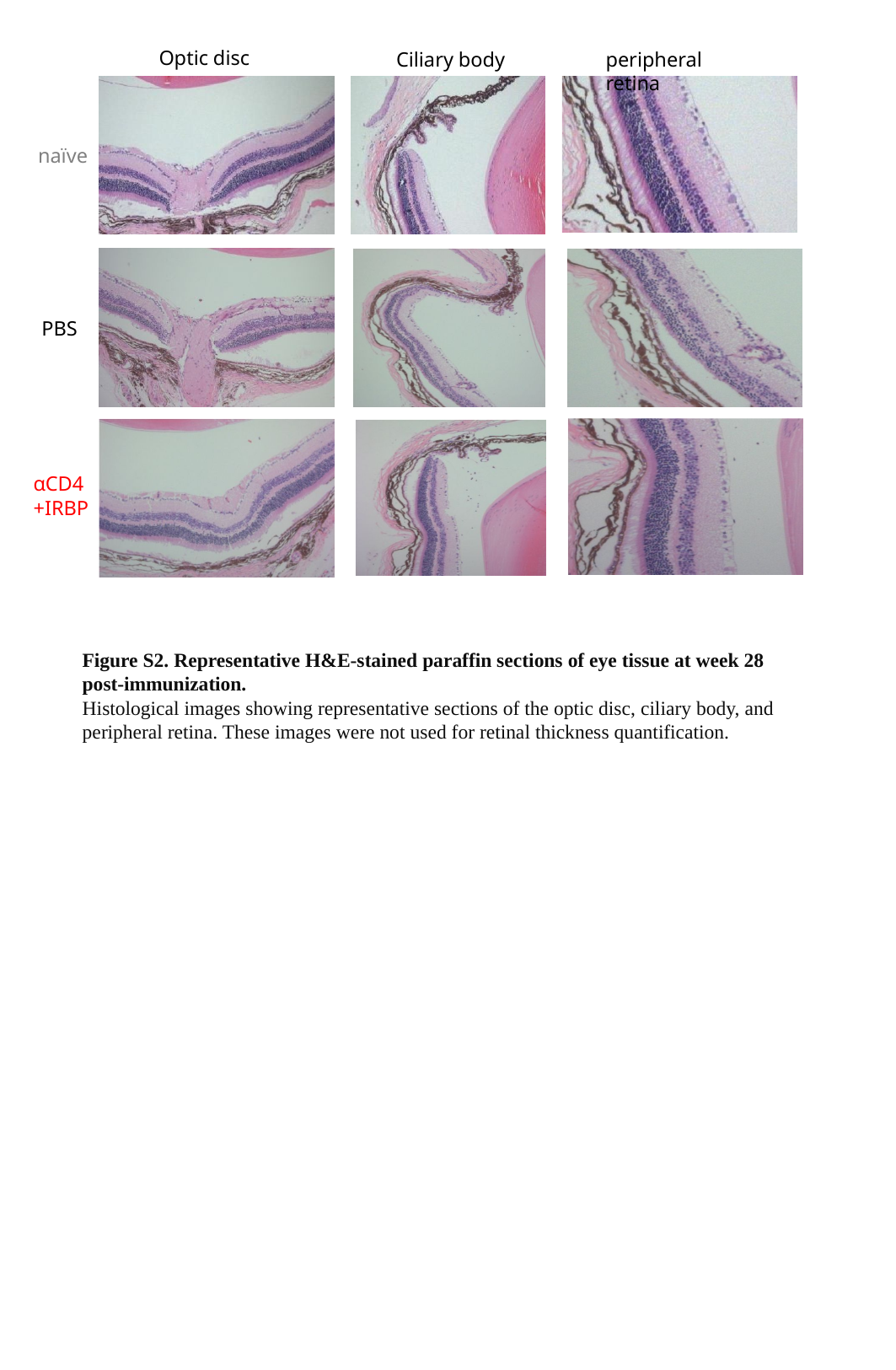

Optic disc
Ciliary body
peripheral retina
naïve
PBS
αCD4
+IRBP
Figure S2. Representative H&E-stained paraffin sections of eye tissue at week 28 post-immunization.
Histological images showing representative sections of the optic disc, ciliary body, and peripheral retina. These images were not used for retinal thickness quantification.

## Slide 3
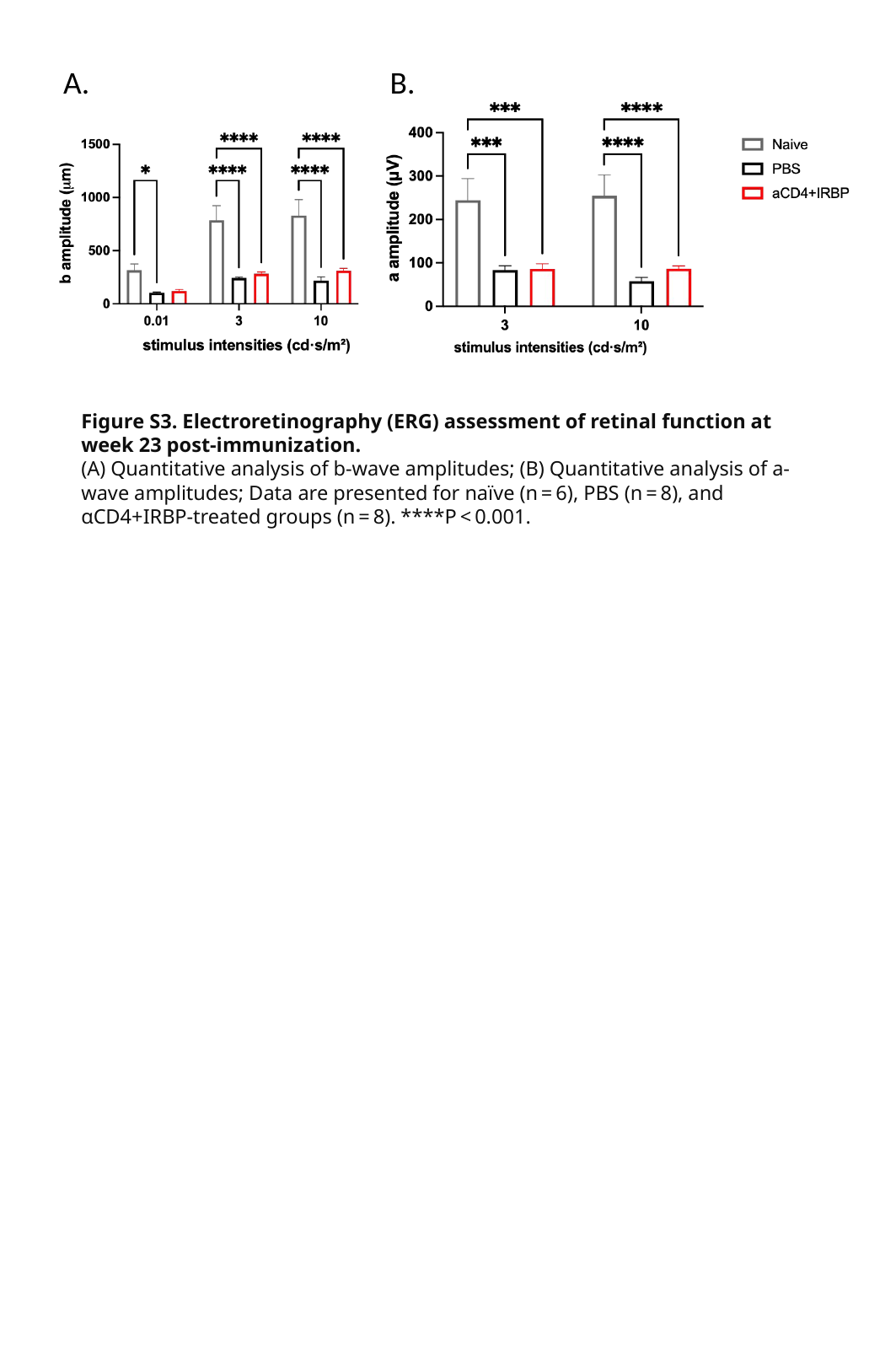

A.
B.
Figure S3. Electroretinography (ERG) assessment of retinal function at week 23 post-immunization.
(A) Quantitative analysis of b-wave amplitudes; (B) Quantitative analysis of a-wave amplitudes; Data are presented for naïve (n = 6), PBS (n = 8), and αCD4+IRBP-treated groups (n = 8). ****P < 0.001.

## Slide 4
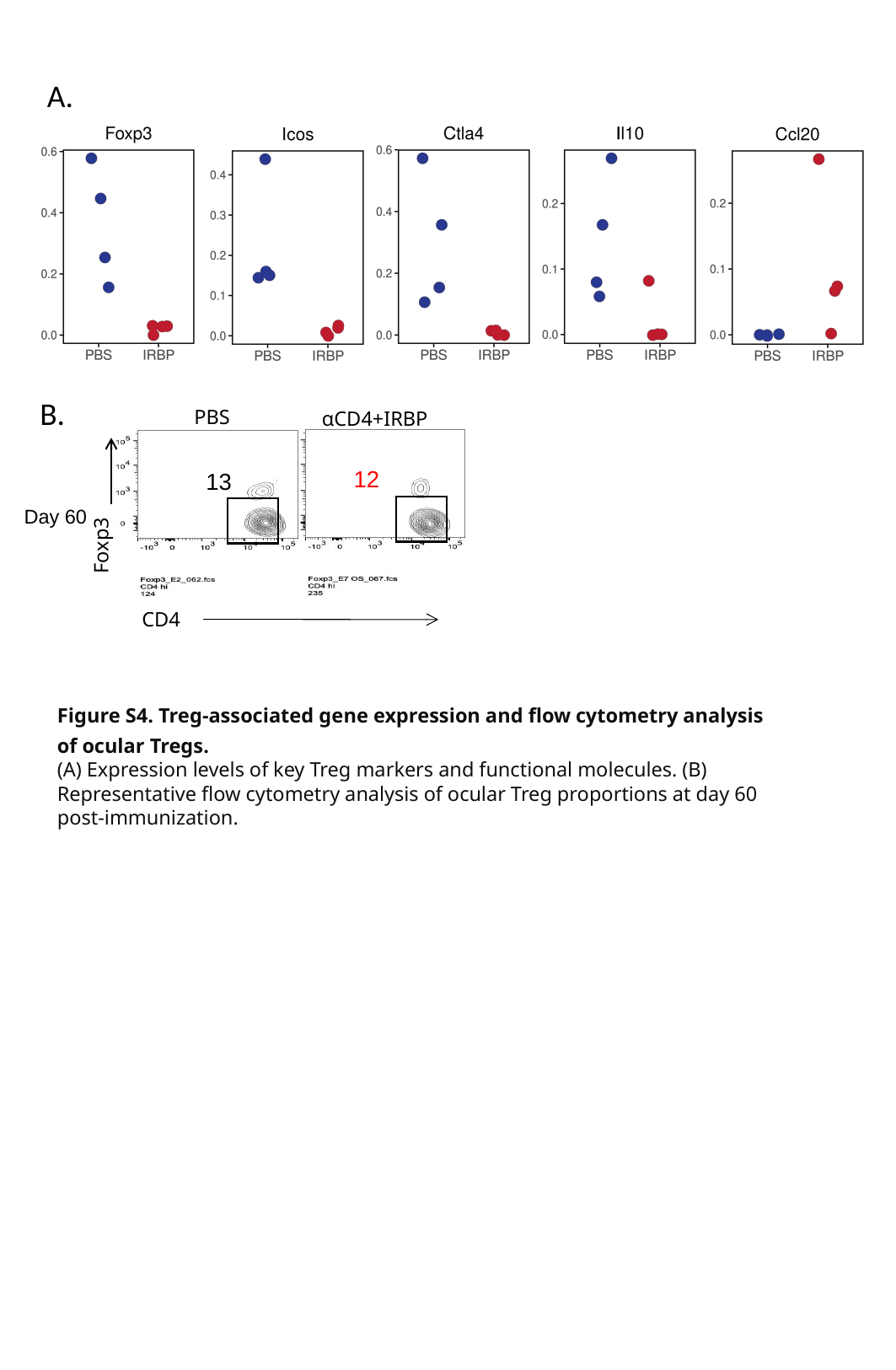

A.
B.
PBS
αCD4+IRBP
Foxp3
CD4
12
13
Day 60
Figure S4. Treg-associated gene expression and flow cytometry analysis of ocular Tregs.
(A) Expression levels of key Treg markers and functional molecules. (B) Representative flow cytometry analysis of ocular Treg proportions at day 60 post-immunization.
